# Chemoenzymatic Synthesis of Select Intermediates and Natural Products of the Desferrioxamine E Siderophore Pathway

**DOI:** 10.3390/molecules27196144

**Published:** 2022-09-20

**Authors:** Katherine M. Hoffmann, Jason S. Kingsbury, Nathan L. March, Yoojin Jang, James H. Nguyen, Miranda M. Hutt

**Affiliations:** Department of Chemistry, Swenson Science Center, California Lutheran University, 60 West Olsen Rd. #3700, Thousand Oaks, CA 91360, USA

**Keywords:** siderophore, NRPS-independent siderophore (NIS) synthesis, *N*-hydroxy-*N*-succinylcadaverine (HSC), ferric chelator, macrocycle, trihydroxamic acid, hydroxamate, bacterial virulence, antibacterial, antibiotic

## Abstract

The NIS synthetase family of enzymes responsible for the biosynthesis of siderophores is increasingly associated with bacterial virulence. Proteins in this class represent outstanding potential drug targets, assuming that basic biochemical and structural characterizations can be completed. Towards this goal, we have mated an improved synthesis of the non-commercial amino acid *N*-hydroxy-*N*-succinylcadaverine (HSC, **6**) with an isothermal titration calorimetry (ITC) assay that profiles the iterative stages of HSC trimerization and macrocyclization by NIS synthetase DesD from *Streptomyces coelicolor*. HSC synthesis begins with multigram-scale Gabrielle and *tert*-butyl *N*-(benzyloxy)carbamate alkylations of 1-bromo-5-chloropentane following prior literature, but the end-game reported herein has two advantages for greater material throughput: (1) hydrogenolysis of benzyl ether and Cbz blocking groups is best accomplished with Pearlman’s catalyst at 40 psi of H_2_ and (2) purification of neutral (zwitterionic) HSC is effected by simple flash chromatography over silica gel in MeOH. HSC is subsequently shown to be a substrate for NIS synthetase DesD, which catalyzes three successive amide bond syntheses via adenyl monophosphate ester intermediates. We quantify and present the iterative and overall enzyme kinetic constants associated with formation of the cyclotrimeric siderophore desferrioxamine E (dfoE, **1**).

## 1. Introduction

**Figure 1:**
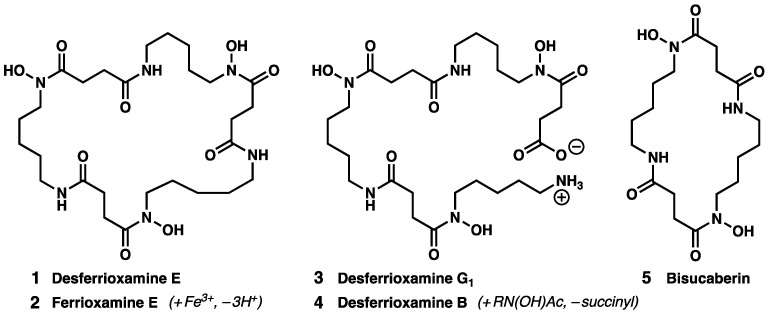
Various hydroxamate ferric-binding siderophores referenced throughout this study.

Siderophores are high-affinity, small molecule, iron chelators [1,2] produced by aerobic and facilitative anaerobic bacteria to compete for and import critical iron ions into the cell [3]. Three functional groups commonly used for ferric iron sequestration are hydroxamates, hydroxycarboxylates, and catecholates [4], which are incorporated into metabolic intermediates or derivatives and linked by amide bonds to form small hexa-dentate chelators [4]. Several hundred variations of siderophores have been isolated, and at times several are attributed to production within the same bacterium. For instance, desferrioxamine E (dfoE, **1**, Figure 1) and G_1_ (dfoG_1_, **3**) are both produced in *Streptomyces* strains [5,6] as well as *Hafnia alvei* and *Erwinia herbicola* in the family Enterobacteriaceae, members of which also produce the catecholate siderophore enterobactin [7]. Additional diversity among the natural products comes in the form of symmetrical dimers such as bisucaberin (**5**) [8,9] and hetero-oligomers like desferrioxamine B (**4**), whose succinyl carboxy terminus is substituted for *N*-hydroxy acetate, precluding macrolactamization. The mesylate salt of dfoB, sold under the trade name Desferal^®^, has seen extensive medical use for the treatment of iron overload, and with a primary amine group (not involved in metal complexation) available for further functionalization, an array of new diagnostic and analytical applications are emerging, including fluorescent sensing [10,11,12].

Among the dominant pathways for siderophore biosynthesis, the NRPS-Independent Siderophore (NIS) synthesis pathway utilizes hydroxamate or carboxylate functions to produce siderophores critical to survival [13,14] and pathogenicity [15,16,17,18,19,20] of bacteria in iron-limiting conditions. This renders enzymes involved in the creation of the virulence factors, specifically the novel NIS synthetases, excellent candidates for inhibition and targeted drug development. NIS synthetases further exhibit a unique protein fold, and are present in at least eighty different isoforms representing at least forty bacteria, many of them pathogenic [14].

**Scheme 1:**
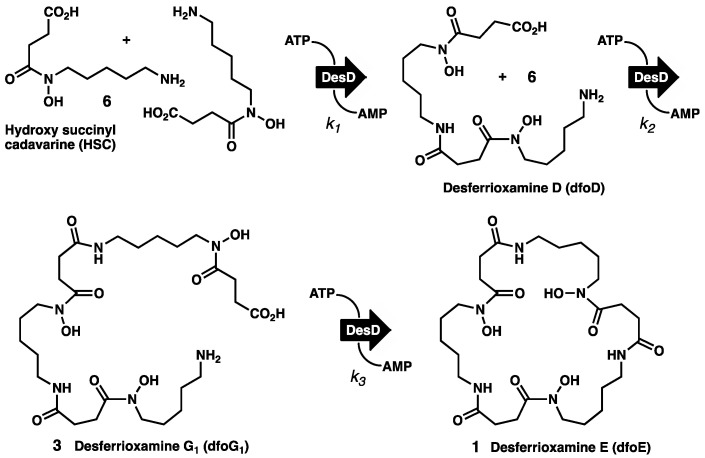
The enzymatic biosynthesis of dfoE (**1**) from HSC (**6**) progresses through dfoD and dfoG_1_ (**3**) as individual condensation products and overall intermediates. DesD is the enzyme catalyst, and ATP is the coupled cofactor that loses pyrophosphate to yield activated AMP esters for acyl transfer.

A sub-population of the family are iterative enzymes, catalyzing multiple condensation reactions on the same substrate (see Scheme 1). The iterative enzymes tend to display broader substrate specificity because of efficient recognition of progressively larger substrates [21,22], and they therefore show greater potential for developing chemotherapeutic agents. A scarcity of basic biochemical research has hampered drug design in this enzyme family, but recent crystal structures and assay developments have jump-started the field [23,24,25,26,27,28,29,30,31,32].

The NIS Synthetase DesD from *Streptomyces coelicolor* is an ideal enzyme model for further inquiry due to the comparably robust literature available [21,22,33,34,35,36]. DesD is an iterative enzyme responsible for forging three successive hydroxamide bonds in dfoE (**1**) between three molecules of *N*-hydroxy-*N*-succinylcadaverine (HSC (**6**), Scheme 1). However, to fully characterize the kinetics and thermodynamics of its iterative catalytic activity [23,33,34,35,36] will require linear amino acid precursors in multiple tether lengths for comparison, and few of the substrates are commercially available. Beyond DesD serving as a model enzyme, studies with other members of the NIS synthetase family—certainly at the level of small molecule protein inhibition—would clearly benefit from more synthesis options related to the derived amino acid reactants.

Only dfoG_1_ (**3**) and dfoE (**1**), the largest substrate and product in the DesD pathway, are commercially available from a single lab in Germany as bacterial isolates. An in vitro biosynthetic approach could create HSC (**6**) from L-lysine, but only if upstream enzymes in the biosynthetic pathway were to be sub-cloned, expressed, and appropriately purified (a decarboxylase, monohydroxylase, and succinase) [22]. While the biosynthesis of dfoE is of interest when characterizing the drug target, a biosynthesis of HSC has the limitations of enzymatic lifetime (short), effort to produce (long) and specificity of substrate recognition, all of which could hamper the design and rapid testing of diverse inhibitors.

A chemical synthesis of HSC would have the advantage of adaptability for future drug and analog accessiblity. Two syntheses [22,37] have been reported as enhancements of an original [38] in which HSC is prepared in seven linear steps involving three protecting groups. Herein, we disclose yet another variation on the total synthesis of HSC in which the final double deprotection is performed under basic conditions and the target molecule is purified over silica gel in neutral form, thus eliminating the need for a gradient HPLC elution reported for the hydrochloride salt of **6** on analytical scales [22]. Additionally, we present a compelling kinetic comparison of HSC versus dfoG_1_ as substrates in DesD’s biosynthesis of dfoE, showcasing the protein’s ability to use varying-sized cognate substrates in its iterative activity.

## 2. Results

### 2.1. Chemical Synthesis of HSC

**Scheme 2:**
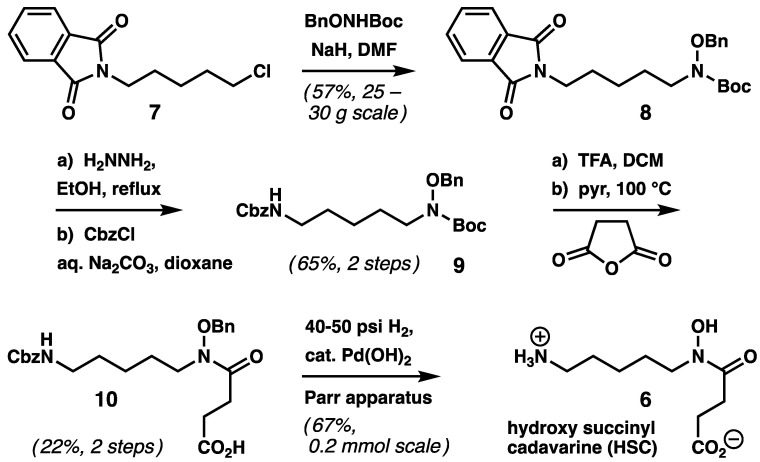
Multigram synthesis of HSC featuring a modified global deprotection and purification.

A practical and scalable pathway to HSC begins with *N*-(benzyloxy)-Boc carbamate anion alkylation of *N*-(5-chloropentyl)phthalimide (**7**) conducted under Finkelstein conditions as previously reported (Scheme 2) [22]. Noteworthy is that **8** can be prepared in similar (60%) efficiency with *N*-(5-bromopentyl)phthalimide as electrophile [37], since it can be purchased from Millipore Sigma. Subsequent exchange of the phthalimide for a Cbz blocking group at the amine terminus occurred as expected, affording differentiated cadaverine **9** in 65% yield over 2 steps after column chromatography. The penultimate *N*-Boc deprotection/succinylation stage of the route continues to present challenges to efficiency given the hindered nature of the secondary amine. Recommended conditions include either 2 h [22] or 30 min [37] of heating succinic anhydride in neat pyridine at 100 °C in the presence of free amine with continued stirring at 23 °C. The purpose of elevated temperature in the forerun is to promote anhydride ring opening by solvent to furnish the more reactive acylpyridinium electrophile for amidation. In our experience, attempts to further optimize the succinylation by changes to times, temperatures, or the use of co-solvents met with failure. However, we also find that following the literature stoichiometry (1.5 equiv succinic anhydride) and heating the reaction mixture for 1 h at 100 °C with recrystallized anhydride in distilled pyridine gave **10** in a reproducible 22% yield over 2 steps. Chromatographic recovery of this material required visualization of fractions with freshly prepared ceric ammonium molybdate (CAM) stain and intense charring of the TLC plates.

Regrettably, the reported conditions for (benzyloxy)carbonyl and benzyloxy ether cleavage (Pd/C, 1 atm of hydrogen), either with [22] or without [37] an HCl additive, did not furnish HSC (**6**) product in our hands when conducted on milligram quantities of **10**. Thus, a more robust protocol was developed and has proven reliable on scales exceeding 0.1 mmole. The procedure substitutes Pearlman’s catalyst (Pd(OH)_2_) for Pd/C and is carried out at elevated hydrogen pressure (40–50 psi) in a Parr shaker apparatus with the original 1:9 *v*/*v* ethyl acetate/*tert*-butanol as solvent [22]. An additional attribute is our finding that analytically pure HSC can be recovered conveniently by flash filtering the concentrated reaction mixture through a silica gel plug with 100% methanol as the eluant. Under these modifications, **6** is isolated in 67% yield as an off-white, foamy solid upon concentration *in vacuo*. Full procedures for the synthesis in Scheme 2 and characterization data for **8**–**10** and **6** are available in Section 4 below and in the Supplementary Materials.

### 2.2. Enzymatic Synthesis of dfoE

**Figure 2:**
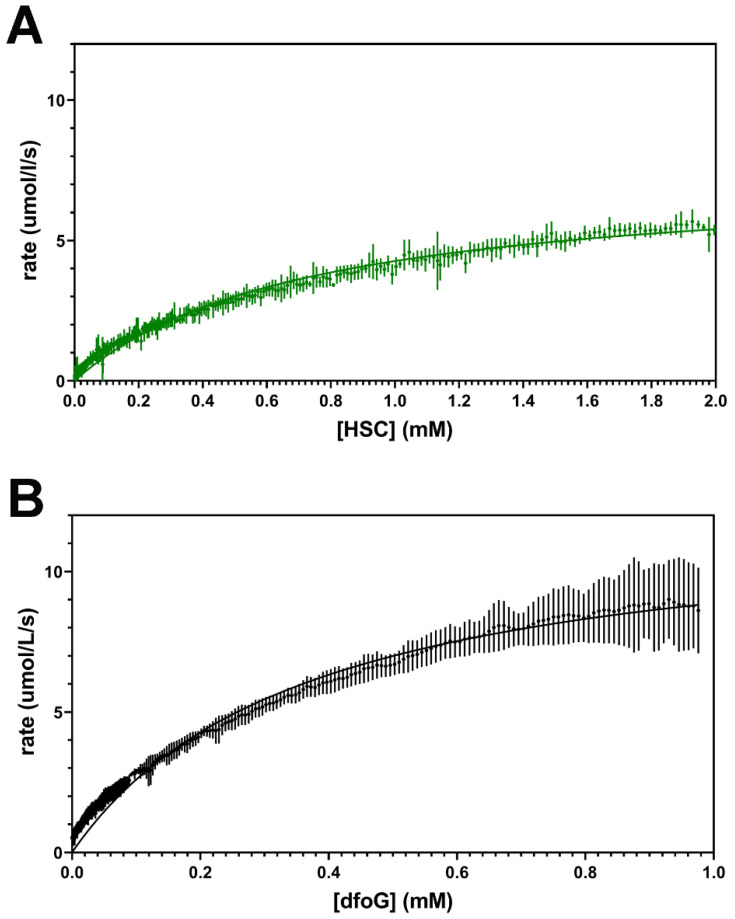
Comparative Michaelis-Menten kinetics of DesD catalytic turnover of dfoE from HSC (**A**, in green) versus dfoG_1_ (**B**, in black, data previously reported [30]). Continuous calorimetric data was ascertained from a single injection of wt DesD at 1 µM concentration into a cell containing 2 mM HSC/10 mM ATP or 1 mM dfoG_1_/5 mM ATP. Each data point represents the average (small cross) and standard deviation (vertical lines) of three replicate trials, and for clarity, every other data point is shown. Data were analyzed in GraphPad Prism 8.2.

Overexpression and purification of DesD produces up to 10 mg of pure protein per liter of cell culture, as previously reported [28]. To utilize synthetic HSC (**6**) prepared as in Scheme 2, we employed a single injection kinetics assay and monitored calorimetric data as three equivalents of HSC react with excess ATP to form dfoE (**1**) (Figure 2A). The velocity versus HSC substrate concentration graphs were well-fit by a non-linear Michaelis-Menten curve. The data reveal a k_cat_ of 7.35 +/− 0.04 s^−1^ and a K_M_ of 0.72 +/− 0.01 mM. The second order rate constant, k_cat_/K_M_ was 10,200 +/− 200 M^−1^ s^−1^, and the ΔH of turnover was −0.4 +/− 0.1 kJ/mol. These findings are summarized in Table 1.

As an iterative enzyme, DesD makes three successive bonds between three molecules of HSC to form dfoE (Scheme 1). To compare the three iterative bond-forming events to the non-iterative, single bond-forming event, we measured HSC kinetics relative to that of the commercially available substrate dfoG_1_ (**3**, previously reported [30], data shown in Figure 2B and Table 1). The k_cat_ for HSC is lower than that of dfoG_1_, by approximately one third. The K_M_, a dissociation constant, is twice that of dfoG1, (where smaller is tighter binding). The secondary rate constant k_cat_/K_M_, sometimes called the specificity constant, is also one third of that for dfoG_1_ within error.

## 3. Discussion

**Figure 3:**
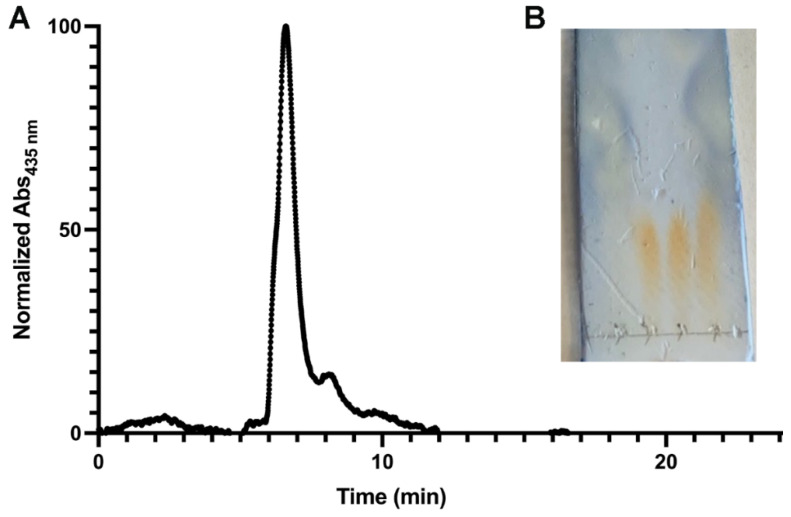
HSC lacks a chromophore, making chromatographic fractions hard to assay. (**A**) Chelation with Fe^3+^ results in a chromophore absorbing at 435 nm, which runs over a reverse phase column in a single peak at 6.6 min. The shoulder at 7.9 min is likely underchelated Fe^3+^ (2:1 or 1:1 ratio). The trace is normalized to the 6.6 min peak. (**B**) A typical TLC photo with CAM staining shows lanes 1 and 5 with higher R*_f_* impurities and splotchy staining; lanes 2, 3, and 4 contain HSC as dark orange.

Some variations in how global deprotection of HSC (**10**→**6**) was performed in prior syntheses (with/without HCl additive), together with the sluggish rate of hydrogenolysis reactions of **10** conducted at dilute concentration under a balloon of H_2_ were the basis for optimization of the procedure detailed in Section 4 below. A switch to Pd(II) hydroxide (Pearlman’s catalyst) ensures a basic reaction medium, and the elevated H_2_ pressure helps saturate the solvent with the gas for smooth double debenzylation. Regarding the former pH issue, final purifications of HSC and its unhydroxylated variant (succinylcadaverine) have previously relied on RP (C18) gradient HPLC assays carried out on the primary ammonium chloride or trifluoroacetate salts [22], steps that require additional considerations for scale-up. Recognizing that deprotected HSC (**6**) would evolve in neutral (zwitterionic) form upon switching to Pd(OH)_2_ as the hydrogenation catalyst, we began experimenting with regular phase silica gel in thin-layer and preparative TLC formats. Ultimately, it was found that non-gradient elution over silica in 100% methanol resulted in facile separation of benzyl alcohol and other greasy impurities from the more polar HSC. Another critical finding related to detection of pure HSC on the stationary phase: while UV light, KMnO_4_, and PMA were totally ineffective, ceric ammonium molybdate (CAM) staining led to dark orange spots of HSC by high-temperature charring of glass-backed TLC plates (Figure 3B).

Synthetic HSC (**6**) has been characterized by ^1^H and ^13^C NMR spectroscopies in deuterium oxide [22] and methanol-*d*_4_ solvents [37], in spite of being less soluble in the latter. Further, diminished solubility of **6** in dry methanol is a benefit to our reported isolation method, as the time course of evaporating clean chromatography fractions results in first cloudiness and later precipitation of **6** as a white solid. On a microscale, it is also possible to triturate methanol or water solutions of **6** with absolute ethanol, a protic solvent it is only sparingly soluble in. Close scrutiny of either D_2_O or CD_3_OD samples of **6** by 500 MHz ^1^H NMR indicates some line broadening and/or shoulder peaks which can be attributed to the presence of discrete hydroxamide rotamers in solution. Characterization data compiled herein are for the predominant rotational isomer, which typically accounts for >90% of the mixture, yet the position of this equilibrium may be temperature-, concentration-, and solvent-dependent. It is interesting to consider whether the presence of amide rotamers has any influence on the kinetics of HSC (and higher siderophore) binding in the active site of DesD, an issue we turn to next in the context of isothermal titration calorimetry (ITC) work carried out with the synthetic small molecule **6** and desferrioxamine G_1_.

The differences in K_M_ (equilibrium dissociation constant for formation of the competent enzyme complex) and k_cat_ (defined for an iterative enzyme as the overall rate of final product formation) are intriguing numbers. The K_M_ for HSC is twice that for dfoG_1_ dissociation, an outcome that may be explained by the stoichiometric requirement that two molecules of HSC (or HSC and dfoD) bind prior to amide bond formation as opposed to a single molecule binding event (for dfoG_1_). Two substrates binding instead of one would suggest an entropic drive in the equilibrium difference. This attractive hypothesis would be best answered by a binding assay that compares the thermodynamics and more deeply probes the impact of iterative behavior on a binding site.

The k_cat_ for HSC is two thirds that of dfoG_1_. This difference may derive from the greater number of HSC molecules (compared to dfoG_1_), and therefore catalytic events, necessary to produce dfoE from HSC. Overall, k_cat_ in this case reflects three sequential events in different active sites that permit priming with the ATP cofactor. Direct comparison of the k_cat_ values is therefore disingenuous—in fact, the dfoG_1_ limiting k_cat_ mirrors k_3_, which is the last rate constant in the sequence of three (Scheme 1). By contrast, the k_cat_ for HSC represents the overall rate of catalysis. A longer term goal is to further distinguish k_1_ and k_2_, which will require a combination of ATP-limiting conditions and dfoD synthesis.

The lower k_cat_ is additionally reflected in the second order rate constant, k_cat_/K_M_. This factor combines the above two constants and is indicative of specificity of rate given the association of a competent complex. The measured three-fold decrease in k_cat_/K_M_ reflecting the triplicate of amide bond mergers may be an excellent comparative tool for further delineation of DesD’s iterative behavior.

Finally, we note that these data were well fit with a Michaelis-Menten curve that did not require any allowance for cooperativity. Previously [30], DesD was hypothesized to be a cooperative enzyme, given its dimerized active sites and iterative behavior. We did not observe this in our prior study of dfoG_1_ kinetics [30], but since that turnover did not reflect the overall k_cat_, it was possible that we would see cooperativity in the present work on HSC kinetics. We now say with confidence that the enzyme does not appear to utilize cooperative kinetics in the overall turnover of dfoE (**1**), an assertion that would not have been possible without the availability of synthetic HSC and of comparison of ITC data.

## 4. Materials and Methods

*General*. All reactions were carried out in flame-dried glassware under an atmosphere of nitrogen in dry and degassed solvent media with standard Schlenk and vacuum-line techniques. Column chromatography was executed with ZEOPrep 60 Eco 40–63 μm silica gel. Analytical thin-layer chromatography (TLC) was performed with 0.25 mm silica gel 60 F254 plates from EMD Chemicals. Spot visualization was accomplished by exposure to UV light and/or staining with ceric ammonium molybdate (CAM) and phosphomolybdic acid (PMA) solutions. Tetrahydrofuran (THF) and dichloromethane (DCM) were stored under argon and dispensed from a solvent purification system manufactured by Pure Process Technologies (Nashua, N.H., USA). Dimethylformamide (DMF) was obtained from Acros in 99.8% anhydrous form (AcroSeal^®^). Hydrazine hydrate and pyridine were distilled over potassium hydroxide and calcium hydride, respectively. *tert*-Butyl *N*-(benzyloxy)carbamate, sodium hydride, benzyl chloroformate, trifluoroacetic acid (TFA), palladium(II) hydroxide, and heptane were obtained from Millipore Sigma and used as received. Succinic anhydride was recrystallized from minimal 1:4 heptane:acetone as thin colorless needles (73% recovery) prior to usage. Absolute ethanol, methanol, petroleum ether, ethyl acetate, and anhydrous diethyl ether were purchased from Pharmco-Aaper in either ACS- or HPLC-grade form for column chromatography.

*Synthesis of t*-*butyl (benzyloxy)(5-(1,3-dioxoisoindolin-2-yl)pentyl)carbamate (**8**)*. Sodium hydride (60% dispersion in mineral oil, 4.944 g, 123.6 mmol, 1.23 equiv) was added to a flame-dried 250 mL round bottom flask containing a Teflon-coated spin bar and washed twice with 50 mL volumes of heptane. In each case, the gray powder was suspended in the hydrocarbon with 30 s of stirring and after settling, the heptane was removed by suction filtration into a syringe using a blunt 20 gauge needle and discarded. The flask was then charged successively with 100 mL of anhydrous DMF by syringe and *tert*-butyl *N*-(benzyloxy)carbamate (25.32 g, 113.4 mmol, 1.13 equiv) in two portions as a solid. A modest gas evolution began, and the resulting gray turbid suspension was heated for 30 min at 85 °C under a positive pressure of nitrogen by submersion in an oil bath. After cooling the reaction mixture to 25 °C, sodium iodide (150.7 mg, 1.006 mmol, 1 mol %) was added as a solid, followed by 2-(5-chloropentyl)-isoindoline-1,3-dione (**7**, 25.31 g, 100.6 mmol) as a solution in 46 mL of DMF (final concentration 0.69 M). This procedure utilized 5-chloro-1-phthalimidopentane [22], but 5-bromo-1-phthalimidopentane can also serve as electrophile and is commercially available from Millipore-Sigma. The resulting cloudy, light yellow reaction mixture was stirred vigorously for 24 h under nitrogen pressure, and **8** was isolated and characterized as described in the Supplementary Materials.

*Synthesis of t-butyl (benzyloxy)(5-(((benzyloxy)carbonyl)amino)pentyl)carbamate (**9**)*. A 250 mL round bottom flask containing a 2.5 cm Teflon stir bar was charged with a solution of **8** (15.55 g, 35.46 mmol, 1.0 equiv) in absolute ethanol (142 mL). With vigorous stirring at 25 °C, hydrazine (5.57 mL, 177.3 mmol, 5 equiv) was added dropwise. Once the addition was complete, the flask was submerged in an oil bath at 80 °C and with continued stirring, a voluminous white and tacky precipitate formed instantaneously throughout the mixture. After 1 h of gentle reflux, the mixture was cooled to 25 °C and poured onto a medium porosity glass frit lined with cotton. The original flask and filter cake were washed with 2 × 30 mL of diethyl ether, and the filtrate was collected directly into another dry 250 mL round bottom flask. Concentration by rotary evaporation gave a yellow oil that was used for subsequent reprotection without purification. The free amine was suspended in 177 mL of 2:1 water: dioxane, with the latter added first to aid in dissolution. With magnetic stirring, sodium carbonate (4.516 g, 42.61 mmol, 1.2 equiv) was added in one portion and the resulting turbid mixture cooled to 0 °C. Finally, benzyl chloroformate (6.08 mL, 42.6 mmol, 1.2 equiv) was added dropwise from a syringe. The cold bath was removed, the reaction mixture was stirred for 24 h, and **9** was isolated by an extractive workup and characterized as described in the Supplementary Materials.

*Synthesis of 4-((benzyloxy)(5-(((benzyloxy)carbonyl)amino)pentyl)amino)-4-oxobutanoic acid (**10**)*. A dry 6 dram vial equipped with a Teflon stir bar and a Teflon-lined screw cap was charged with a solution of **9** (509 mg, 1.15 mmol) in 9.2 mL of dichloromethane. With stirring, the solution was cooled to 0 °C and treated dropwise with 2.3 mL (30 mmol, 0.1 M final conc.) of trifluoroacetic acid (TFA), at which point the mixture turned yellow. The ice bath was removed and stirring continued at 25 °C for 3 h. The dichloromethane was then purged off under a stream of nitrogen with constant stirring in a lukewarm water bath, and the excess TFA was removed under high vacuum—first at 0 °C to permit degassing and to prevent bumping. The resulting light orange oil (quant. yield) showed characterization data matching the literature [22] and was used without further purification. Anhydrous pyridine (3.8 mL, 0.3 M) and recrystallized succinic anhydride (177 mg, 1.77 mmol, 1.54 equiv) were then added in succession to the vial. With stirring at 25 °C, the free amine immediately dissolved, yet crystals of the anhydride stayed suspended. The vial was purged with nitrogen, secured tightly with its screw cap, and submerged into a pre-heated oil bath for 1 h of stirring at 100 °C. The resulting homogeneous solution was removed from the hot bath and stirring was continued for 24 h at ambient temperature. **10** was isolated and characterized as described in the Supplementary Materials.

*Deprotection and Purification of N-hydroxy-N-succinylcadaverine (HSC, **6**)*. A tall reaction chamber standard to a Parr shaker apparatus/hydrogenator was charged with a solution of 66.4 mg (0.150 mmol, 1.0 equiv) of 4-((benzyloxy)(5-(((benzyloxy)carbonyl)amino)pentyl)amino)-4-oxobutanoic acid (**10**) in 5.3 mL of *t*-butanol (Aldrich) and further diluted with 0.7 mL of ethyl acetate (9:1 *v*/*v t*-BuOH/EtOAc, 0.025 M). Pd(OH)_2_ (10.5 mg, 0.0150 mmol based on a 20 wt% loading, 0.10 equiv) was then added as a solid, and the vessel was sealed with the integrated cap assembly (with a Teflon tube connected to the Parr’s hydrogen chamber). Three sets of evacuate/purge cycles were then applied to the reaction mixture using a small diaphragm pump to apply negative pressure. Finally, a positive pressure exceeding 40 psi of H_2_ was applied to the flask and the mixture was agitated on the shaker for 12 h at 25 °C. The black suspension was then filtered through cotton and Celite to remove Pearlman’s catalyst, and the filtrate was concentrated to a light yellow oil that showed complete consumption of starting material by TLC and benzyl alcohol as the primary contaminant by ^1^H NMR analysis. Purification by silica gel chromatography with 100% HPLC-grade methanol for both loading and elution furnished 21.9 mg (67% yield) of **6** (R*_f_* = 0.40) as a colorless, amorphous solid after rotary evaporation of pure fractions with a belt-driven vacuum pump and further drying *in vacuo* (< 1 mmHg). A photograph (Figure 3B) was included (Section 3) to emphasize the requirement for fresh CAM stain and high-temperature charring of TLC plates (with an industrial heat gun) in order to visualize the dark orange-brown, streaky spots of HSC. Characterization data matched those in the literature [22,37]. ^1^H NMR (D_2_O, 500 MHz) δ 3.68 (t, *J* = 6.6 Hz, 2H), 3.02 (t, *J* = 7.3 Hz, 2H), 2.76 (t, *J* = 7.1 Hz, 2H), 2.50 (t, *J* = 7.1 Hz, 2H), 2.48 (s, 1H), 1.66–1.76 (m, 4H), 1.39 (quintet, *J* = 7.6 Hz, 2H); ^13^C NMR (CD_3_OD, 125 MHz) δ 181.0, 175.4, 47.6, 40.5, 34.3, 29.3, 27.9, 26.8, 24.0; HRMS (ESI+) Calcd. for C_9_H_19_N_2_O_4_^+^ [M+H]^+^: 219.1345, Found 219.1344. Purity was monitored before use in biochemical assays by chelation with freshly prepared FeCl_3_ in acetonitrile (1:10 ratio, orange solution, peak absorbance 340 nm), which resulted in a dark red solution having a peak absorbance at 435 nm [7] that was assayed by reverse phase HPLC (4 mm x 250 mm Prontosil column, 8% acetonitrile in 0.5% acetic acid running buffer, flow rate 0.5 mL/min; HSC elutes at 6.6 min with a small shoulder at 7.9 min). Control runs of FeCl_3_ have a trace absorbance at 7.9 min as well, assigned as free or under chelated Ferric iron in solution (see Figure 3A, Section 3).

*Expression and Purification of Recombinant Enzymes*. Expression, purification, storage and quantification of DesD was as described previously [30]. Briefly, crude homogenates of overexpressed protein were purified to homogeneity by nickel affinity chromatography (NiNTA) using an ÄKTA start FPLC (Cytiva, Cardiff, Wales). All proteins were dialyzed into ITC buffer overnight and quantified by UV/Vis chromatography at 280 nm using calculated extinction coefficient.

*Steady State Kinetics Methods*. All ITC experiments were performed at 37 °C, in 50 mM HEPES buffer at pH 7.5, containing 150 mM NaCl, 5 mM TCEP, 15 mM MgCl_2_, and 25% glycerol. A single injection of enzyme into a substrate mixture containing 1–2 mM substrate (HSC or dfoG_1_) and either 5 or 10 mM ATP was monitored continuously for differences in thermal power as described previously [30]. The raw data thermogram reflects a reaction where the substrate is completely consumed, allowing the calculation of total Q, and consequently, the total enthalpy change in the calorimeter (ΔH_app_). Where V is the cell volume (~280 µL), the change in heat is proportional to rate as in Equation (1) below:

(1) $$Rate\;(\vartheta )=\frac{1}{V*\;\Delta H_{app}}*\;\frac{dQ}{dt}$$

Analysis was performed using GraphPad Prism 8.2, where V_max_ = k_cat_ ∗ [E] and the data were well-fit with one of the non-linear regressions, below (Equation (2)), to calculate k_cat_ and K_M_ values for cooperative or non-cooperative kinetics.

(2) $$\vartheta =\frac{V_{max}*\;\left[S\right]}{K_{M}*\;\left[S\right]}$$

## 5. Conclusions

We have presented a variation on the total synthesis of HSC in which the final double deprotection is performed efficiently under basic conditions and the target molecule is purified over silica gel in neutral form. This has permitted the first compelling kinetic comparison of HSC (monomer) versus dfoG_1_ (trimer) as substrates in DesD’s biosynthesis of dfoE, showcasing the ability to utilize varying-sized cognate substrates in its iterative activity. This has permitted the first comparative analysis of the iterative turnover with a single step, and it shows that the primary rate-limiting step is association rather than turnover. Given the fact that macrocycle formation remains a significant challenge in organic synthesis, we find the merger of chemical and enzymatic syntheses to be a convenient strategy for accessing a range of siderophore precursors and NIS synthetase inhibitors that could represent lead structures towards brand new classes of antibiotics.

## Figures and Tables

**Table 1:** Reported kinetic constants for DesD with the limiting substrate identity in parentheses.

	kcat (s^−1^)	K_M_ (mM)	k_cat_/K_M_ (M^−1^ s^−1^)	ΔH (kJ/mol)
DesD (HSC)	7.35 +/− 0.04	0.72 +/− 0.01	10,200 +/− 200	−0.4 +/− 0.1
DesD (dfoG_1_)	11.1 +/− 0.2	0.35 +/− 0.01	32,000 +/− 1000	−0.9 +/− 0.1

## Data Availability

Data presented in this study are available in the form of procedures given in Section 3, in the Supplemental Material, or from the corresponding author upon request.

## Supplementary Materials

The following supporting information can be downloaded at: https://www.mdpi.com/article/10.3390/molecules27196144/s1.

## Sample Availability

Samples of synthetic HSC (in milligram quantities) and of intermediates **7**–**10** (in multi-milligram quantities) are available by request to J.S.K. for authenticity or experimentation.
