# Supplementary material for: Chemoenzymatic Synthesis of Select Intermediates and Natural Products of the Desferrioxamine E Siderophore Pathway
Source: Molecules. 2022 Sep 20;27(19):6144. doi: 10.3390/molecules27196144 (PMC9571020; doi:10.3390/molecules27196144)

Chemoenzymatic Synthesis of Select Intermediates and Natural Products of the Desferrioxamine E Siderophore Pathway.

Katherine M. Hoffmann*, Jason S. Kingsbury, Nathan L. March, Yoojin Jang, James H. Nguyen, and Miranda M. Hutt

Department of Chemistry, Swenson Science Center, California Lutheran University, 60 West Olsen Rd. #3700, Thousand Oaks, CA, 91360, U.S.A.

General. Infrared spectra were recorded on an FT-IR spectrophotometer; peaks are reported in units of wavenumbers (cm^{-1}) as strong (s), medium (m), weak (w), and broad (br). ^{1}H NMR data were recorded on a 500 MHz instrument at California State University, Channel Islands (CSUCI) (Camarillo, CA) and are reported as follows: chemical shift, multiplicity (s = singlet, d = doublet, t = triplet, q = quartet, br = broad, m = multiplet), *J* values (Hz), and integration. ^{1}H and ^{13}C shifts are reported in ppm from tetramethylsilane with the solvent resonance as the internal standard. High-resolution mass spectra (HRMS) were obtained using Data Acquisition in Real Time (DART) with ESI-TOF at the Mass Spectrometry Laboratory in the School of Chemical Sciences at the University of Illinois (Urbana, IL).

Purification of *tert*-butyl (benzyloxy)(5-(1,3-dioxoisoindolin-2-yl)pentyl)carbamate (8). The reaction mixture was diluted with 100 mL of cold DI water and 75 mL of diethyl ether and transferred with rinsing to a 500 mL separatory funnel. After agitation, the light yellow organic layer was removed and the colorless aqueous layer was washed three times with 75, 50, and 50 mL portions of ether. In the same 500 mL separatory funnel, the combined organic layers were washed 3 x with 200 mL portions of saturated aqueous sodium chloride to remove residual DMF. The extract was dried over $MgSO_4$ and filtered through a medium porosity glass frit into a 500 mL round bottom flask, at which point concentration by rotary evaporation afforded a viscous bronze oil that was purified by flash chromatography with 7:1 petroleum ether:ethyl acetate ($R_f = 0.35$) as eluant and PMA (or potassium permanganate) stain for spot visualization. Concentration of pure fractions afforded (25.05 g, 57%) of the desired product **8** as a colorless oil. Characterization data was consistent with prior literature.[1] IR (neat, cm^{-1}) 3029, 2930, 1707, 1393, 1364, 1155; ^{1}H NMR ($CDCl_3$, 500 MHz) δ 7.83 (dd, *J* = 5.2, 3.1 Hz, 2H), 7.70 (dd, *J* = 5.5, 3.1 Hz, 2H), 7.30-7.40 (m, 5H), 4.81 (s, 2H), 3.66 (t, *J* = 7.3 Hz, 2H), 3.38 (t, *J* = 7.3 Hz, 2H), 1.67 (quintet, *J* = 7.6 Hz, 2H), 1.62 (quintet, *J* = 7.6 Hz, 2H), 1.49 (s, 9H), 1.29-1.36 (m, 2H); ^{13}C NMR ($CDCl_3$, 125 MHz) δ 168.5, 156.7, 135.8, 134.0, 132.3, 129.5, 128.6, 128.5, 123.3, 81.3, 77.0, 49.6, 38.0, 28.4, 26.8, 24.2. HRMS (ESI+) Calcd. for $C_{25}H_{31}N_2O_5^+$ $[M+H]^+$: 439.2227, Found 439.2223. Anal. Calcd. for $C_{25}H_{30}N_2O_5$: C, 68.47, H, 6.90; Found C, 68.35, H, 6.91.

(1) See Supplementary Information for ref. 22 and Supporting Information for ref. 37 of this article for comparison.

Purification of *t*-butyl (benzyloxy)(5-(((benzyloxy)carbonyl)amino)pentyl)carbamate (9). The Cbz protection mixture was diluted with 100 ml of dichloromethane and poured over 80 mL of saturated aq. sodium chloride in a 500 mL separatory funnel. After extraction and removal of the organic layer, the aqueous layer was washed 2x with 75 mL of dichloromethane. The combined organic layers were dried over $MgSO_4$, filtered, and concentrated to a crude, viscous oil. Purification was achieved by chromatography over silica gel using 5:1 petroleum ether:ethyl acetate as eluant ($R_f = 0.40$) and a CAM stain for product visualization. Concentration of pure fractions afforded 10.2 g (65%) of **9** as a colorless oil that showed characterization data consistent with prior literature.[1] IR (neat, cm^{-1}) 3350, 2993, 1693, 1524, 1448, 1362, 1240; ^1H NMR ($CDCl_3$, 500 MHz) δ 7.29-7.41 (m, 10H), 5.09 (s, 2H), 4.81 (s, 2H), 4.73 (br s, 1H), 3.40 (t, *J* = 7.0 Hz, 2H), 3.17 (dd, *J* = 13, 7.0 Hz, 2H), 1.56-1.62 (m, 2H), 1.47-1.53 (m, 2H), 1.49 (s, 9H), 1.26-1.32 (m, 2H); ^{13}C NMR ($CDCl_3$, 125 MHz) δ 156.5, 156.3, 136.5, 135.5, 129.3, 128.4, 128.3, 128.1, 128.0, 127.9, 81.1, 76.8, 66.4, 49.2, 40.8, 29.4, 28.2, 26.6, 23.7. Anal. Calcd. for $C_{25}H_{34}N_2O_5$: C, 67.85, H, 7.74; Found C, 67.76, H, 7.81.

Purification of 4-((benzyloxy)(5-(((benzyloxy)carbonyl)amino)pentyl)amino)-4-oxobut-anoic acid (10). After Boc removal and succinylation, the stir bar was rinsed with 0.2 mL of chloroform, removed from the vial, and pyridine was removed on a rotary evaporator mated with a belt-driven high vacuum pump (~1 mm Hg). The resulting amber oil was purified by silica gel chromatography in 9:1 dichloromethane:methanol ($R_f = 0.50$). Fractions were carefully assayed for product using a freshly prepared CAM stain. Owing to the acid group, the product streaks on the stationary phase; similar results are observed with 100% ethyl acetate or diethyl ether as eluants, and a trailing impurity appears to aggregate or co-elute with the product. Only fractions giving an intense, dark brown leading edge to the comet-like spot upon charring with CAM were collected as pure material. Concentration of such fractions afforded 113 mg (22% yield) of desired product as a yellow semi-solid. Characterization data was consistent with the literature.[1] IR (neat, cm^{-1}) 3061, 2934, 1707, 1653, 1453, 1242; ^1H NMR ($CDCl_3$, 500 MHz) δ 7.26-7.40 (m, 10H), 5.10 (s, 2H), 4.94 (br s, 1H), 4.81 (s, 2H), 3.62-3.74 (m, 2H), 3.10-3.20 (m, 2H), 2.70 (t, *J* = 6.5 Hz, 2H), 2.63 (t, *J* = 6.5 Hz, 2H), 1.60-1.68 (m, 2H), 1.42-1.53 (m, 2H), 1.23-1.35 (m, 2H); ^{13}C NMR (CD_3OD, 125 MHz) δ 176.2, 176.0, 158.8, 138.4, 136.1, 130.5, 129.8, 129.6, 129.3, 128.8, 128.6, 77.1, 67.2, 46.0, 41.5, 30.3, 29.2, 28.3, 27.3, 24.6. HRMS (ESI+) Calcd. for $C_{24}H_{31}N_2O_6^+$ $[M+H]^+$: 443.2182, Found 443.2178. Anal. Calcd. for $C_{24}H_{30}N_2O_6$: C, 65.14, H, 6.83; Found C, 65.35, H, 6.91.

4-((5-aminopentyl)(hydroxy)amino)-4-oxobutanoic acid (6). Conditions for Pd-catalyzed hydrogenolysis of the Cbz and Bn blocking groups to furnish hydroxy succinyl cadaverine (HSC, **6**) are provided in section 4 (Materials and Methods) of the article along with characterization data.

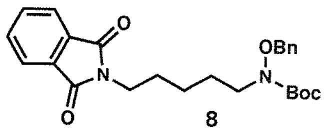

8

Ally-amine

Sample Name	**Ally-amine**	Pulse sequence	**PROTON**	Temperature	**24**	Study owner	**walkup**
Date collected	**2022-05-26**	Solvent	**cdcl3**	Spectrometer	**wormhole-vnmrs500**	Operator	**walkup**

PEAK FREQUENCIES

index	freq(ppm)	intensity
1	7.83538	14.1834
2	7.82462	12.6564
3	7.81777	15.5108
4	7.70524	18.0904
5	7.69937	14.9027
6	7.69447	14.2583
7	7.6886	15.0643
8	7.38428	13.8372
9	7.38134	13.6491
10	7.34415	16.3867
11	7.33046	13.4911
12	7.26	13.5983
13	4.80679	46.4551
14	3.66189	18.5993
15	3.38594	13.9306
16	1.48659	268.199
17	-0.00471344	212.523

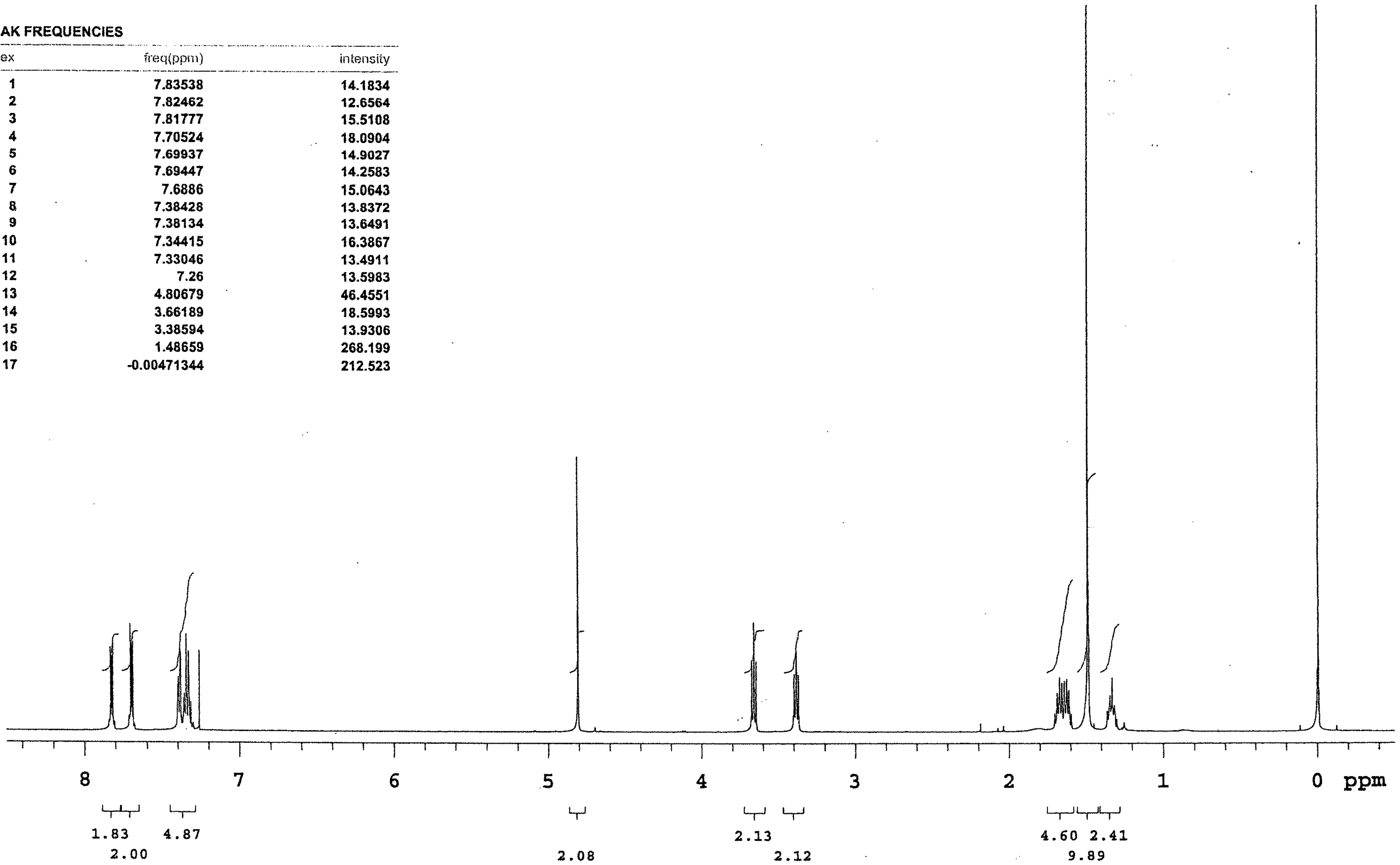

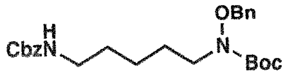

9

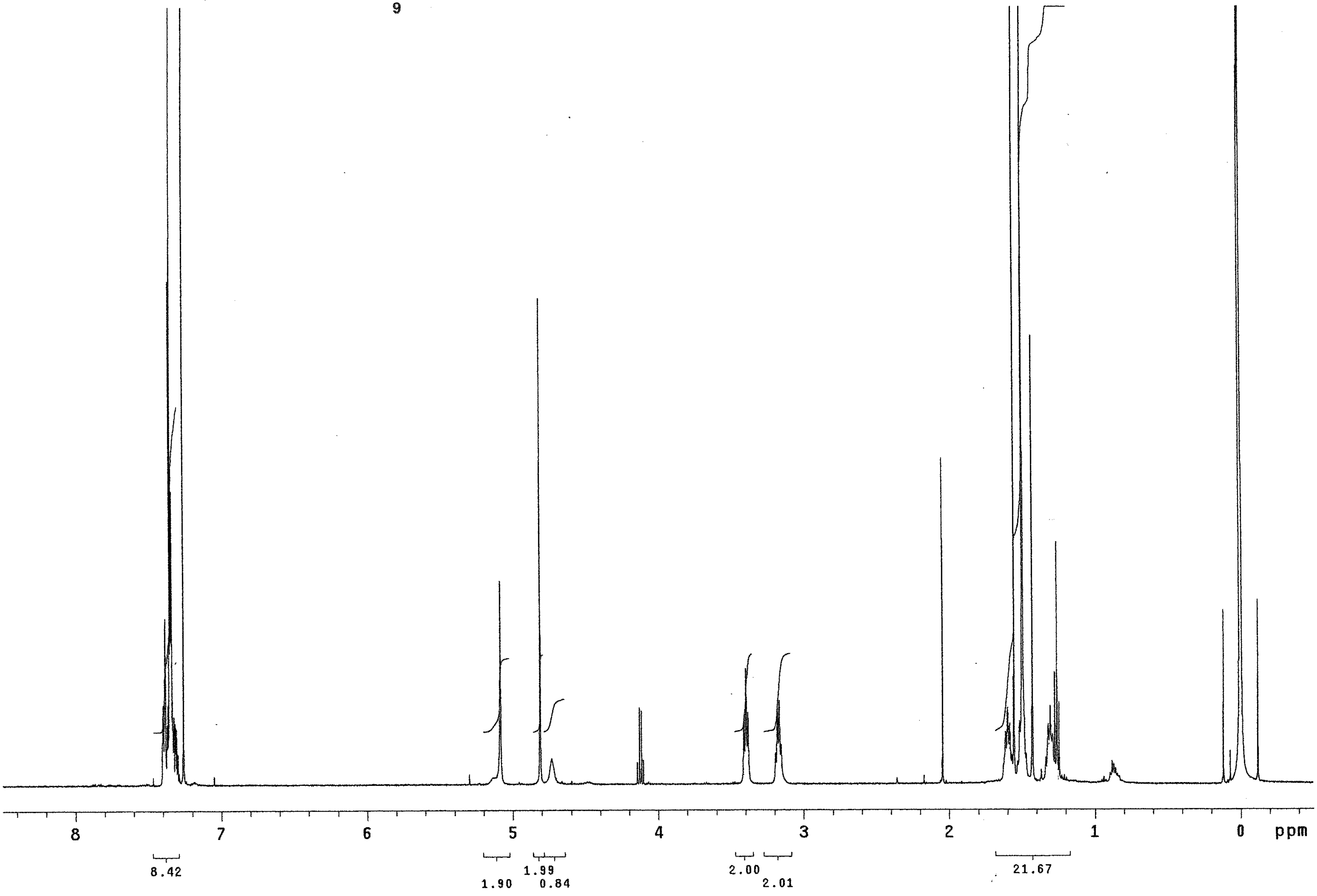

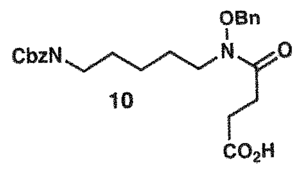
OBn
CbzN
N
O
10
CO2H

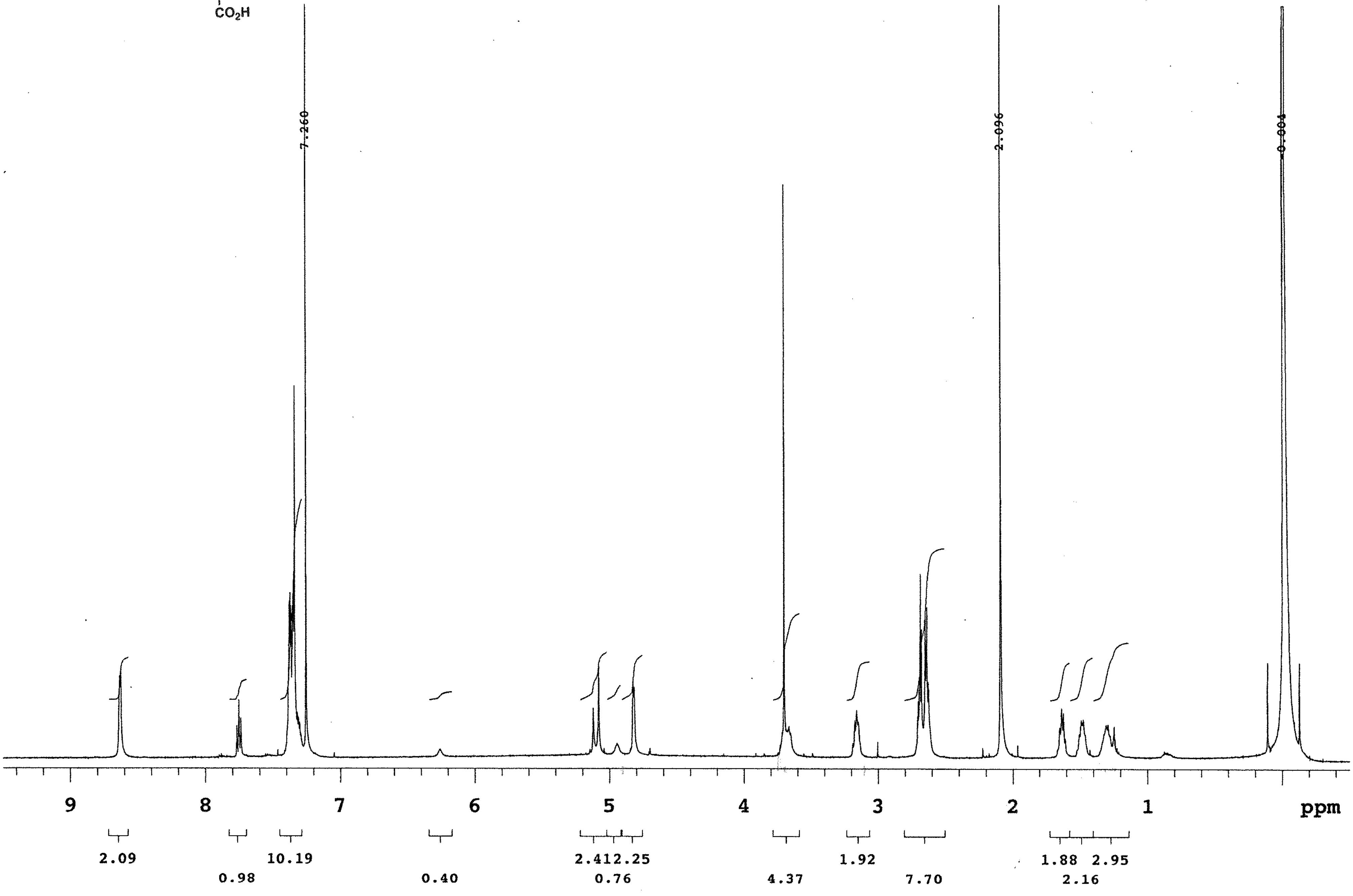
7.260
2.096
-0.004
9
8
7
6
5
4
3
2
1
ppm
2.09
0.98
10.19
0.40
2.412.25
0.76
4.37
1.92
7.70
1.88 2.95
2.16

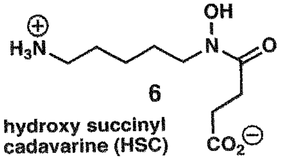

6

hydroxy succinyl cadavarine (HSC)

INTEGRAL VALUES

Integral	start(ppm)	end	value
1	3.72329	3.63942	1.647
2	3.09177	2.98323	2.000
3	2.81055	2.71187	1.554
4	2.55893	2.44052	2.705
5	1.82873	1.63138	4.276
6	1.46857	1.28602	2.371

PEAK FREQUENCIES

index	freq(ppm)	intensity
1	4.79	20711.2

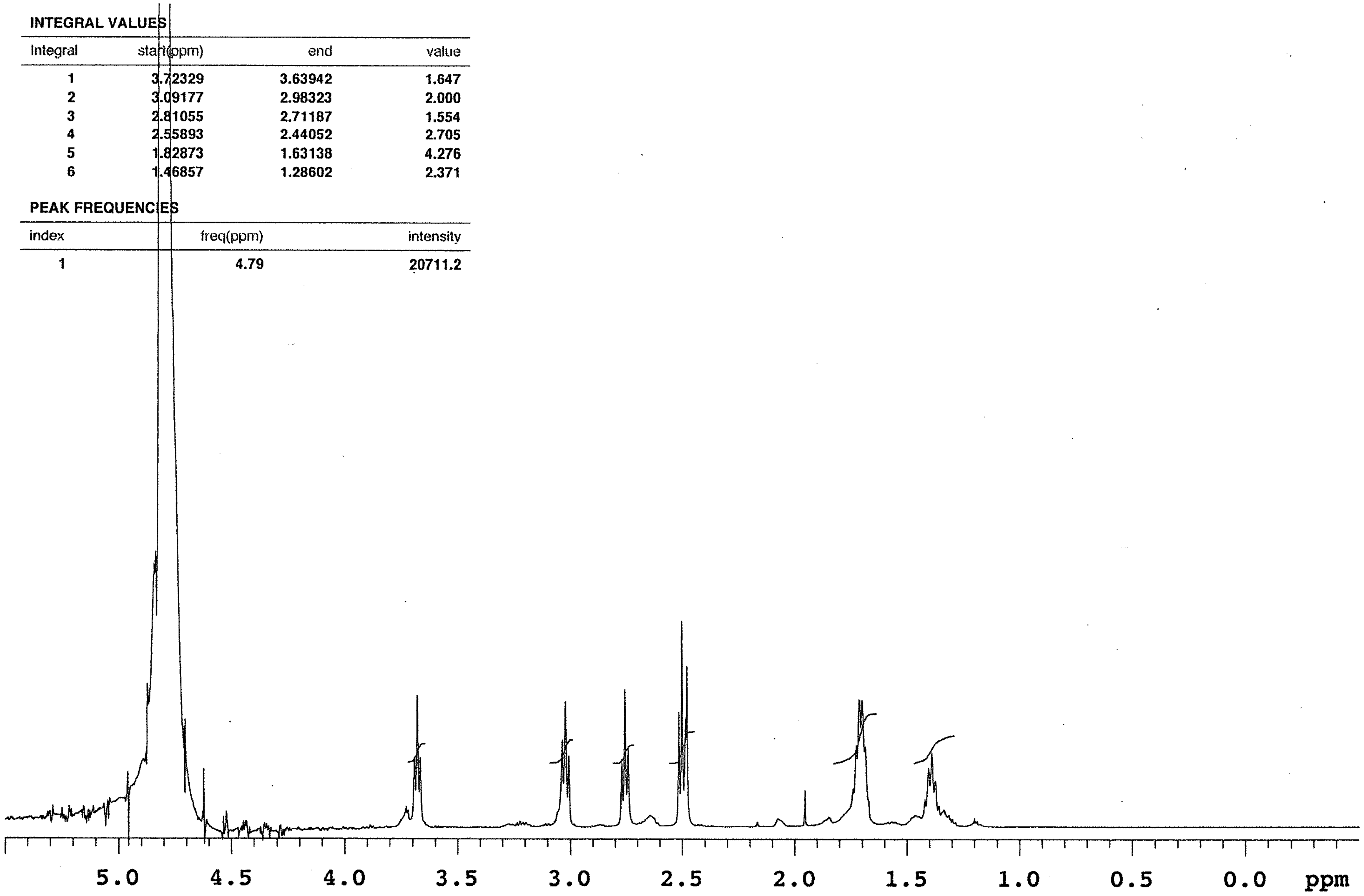

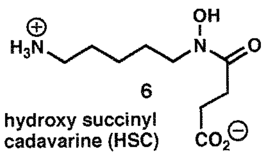

PEAK FREQUENCIES

index	freq(Hz)	intensity
1	1845.26	30.7965
2	1838.41	59.1107
3	1832.05	30.8649
4	1517.09	38.1682
5	1509.76	56.0652
6	1502.42	31.7076
7	1384.07	27.7973
8	1377.22	62.0835
9	1369.89	35.4585
10	1256.91	51.5594
11	1250.07	93.0549
12	1242.73	48.7215
13	1238.82	72.3936
14	862.727	36.2321
15	856.369	57.3826
16	849.522	56.8218
17	842.675	35.7897
18	702.314	26.0334
19	694.978	33.1545
20	687.153	20.4164

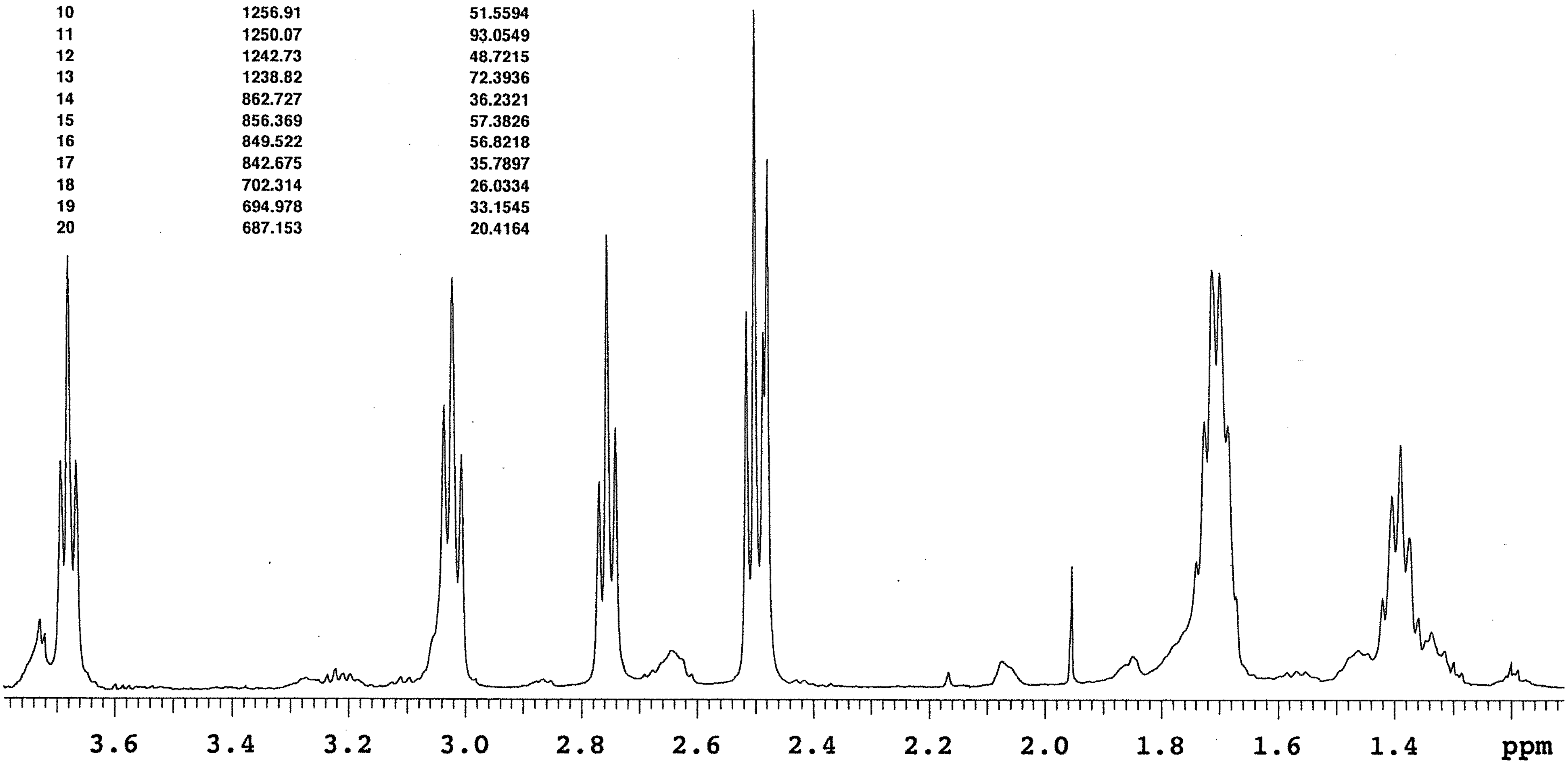

Supplement: Supplementary file 1 [file molecules-27-06144-s001.zip › molecules-1898168-supplementary.pdf]
